# THE BLACK BOX OF AI-AGEISM: EXPLORING THE CO-CONSTITUTION OF AGING AND ARTIFICIAL INTELLIGENCE

**DOI:** 10.1093/geroni/igae098.1438

**Published:** 2024-12-31

**Authors:** Vera Gallistl, Muneeb Ul Lateef Banday, Clara Berridge, Alisa Grigorovich, Juliane Jarke, Ittay Mannheim, Barbara Marshall, Alexander Peine

**Affiliations:** Karl Landsteiner University of Health Sciences, Krems, Niederosterreich, Austria; University of Bern, Bern, Bern, Switzerland; University of Washington, Seattle, Washington, United States; Brock University, St. Catharines, Ontario, Canada; BANDAS Centre, University of Graz, Graz, Steiermark, Austria; Ben Gurion University of the Negev, Beer-Sheva, HaDarom, Israel; Trent University, Toronto, Ontario, Canada; Open University of the Netherlands, Heerlen, Limburg, Netherlands

## Abstract

Algorithmic technologies and (large) data infrastructures, often referred to as Artificial Intelligence (AI), have received increasing attention from gerontological research in the last decade. So far, however, available studies have often focused on evaluating the “impact” of AI technologies on the lives of older people, while critical engagement with theory has been largely absent in these studies. In this paper, we make a novel contribution by critically engaging with the theorizing in this growing field of research. We observe that gerontology’s engagement with AI is shaped by an interventionist logic that situates AI as a black box for gerontological research. We demonstrate how this black box logic has neglected many aspects of AI as a research topic for gerontology and discuss three classical concepts in gerontology to show how they can be used to open various black boxes of aging and AI in the areas: a) the datafication of aging, b) the political economy of AI and aging, and c) everyday engagements and embodiments of AI in later life. In the final chapter, we propose a model of the co-constitution of aging and AI that makes theoretical propositions to study the relational terrain between aging and AI and hence aims to open the black box of AI in gerontology beyond an interventionist logic.

